# Human-like Decision-Making System for Overtaking Stationary Vehicles Based on Traffic Scene Interpretation

**DOI:** 10.3390/s21206768

**Published:** 2021-10-12

**Authors:** Jinsoo Yang, Seongjin Lee, Wontaek Lim, Myoungho Sunwoo

**Affiliations:** 1 Department of Automotive Engineering, Hanyang University, Seoul 04763, Korea; jsyang1124@gmail.com; 2 ACELAB, 13, Eonju-ro 81-gil, Gangnam-gu, Seoul 06222, Korea; seongjin90@gmail.com (S.L.); lwt1849@gmail.com (W.L.); 3 Department of Automotive Convergence, College of Engineering, Korea University, Seoul 02841, Korea

**Keywords:** motion planning, maneuver planning, behavioral planning, human-like decision-making, overtake stationary vehicles, discretionary lane change, overtaking maneuver decisions

## Abstract

There are multifarious stationary vehicles in urban driving environments. Autonomous vehicles need to make appropriate overtaking maneuver decisions to navigate through the stationary vehicles. In literature, overtaking maneuver decision problems have been addressed in the perspective of either discretionary lane-change or parked vehicle classification. While the former approaches are prone to generating undesired overtaking maneuvers in urban traffic scenarios, the latter approaches induce deadlock situations behind a stationary vehicle which is not distinctly classified as a parked vehicle. To overcome the limitations, we analyzed the significant decision factors in the traffic scenes and designed a Deep Neural Network (DNN) model to make human-like overtaking maneuver decisions. The significant traffic-related and intention-related decision factors were harmoniously extracted in the traffic scene interpretation process and were utilized as the inputs of the model to generate overtaking maneuver decisions in the same manner with the human driver. The overall validation results convinced that the extracted decision factors contributed to increasing the learning performance of the model, and consequently, the proposed decision-making system enabled the autonomous vehicles to generate more human-like overtaking maneuver decisions in various urban traffic scenarios.

## 1. Introduction

**Figure 1:**
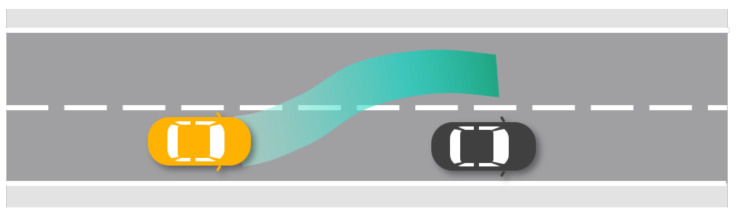
Overtake a stationary vehicle on road.

With the advancement of autonomous driving technologies, various decision-making systems for driving maneuvers have been developed to navigate in dynamic environments [1,2,3,4,5]. Nevertheless, many challenges are remaining in handling unexpected situations in urban environments. One of the challenges is in making overtaking maneuver decisions when the autonomous vehicle encounters stationary vehicles as shown in Figure 1. A decision-making problem is commonly divided into two parts. The first part is to determine what kinds of information to be considered in decisions and the second part is to establish a methodology to derive a decision from the selected factors. Since stationary vehicles on urban roads may occur in multifarious kinds of situations, it is necessary to thoroughly examine important decision factors and select an appropriate strategy for overtaking maneuver decisions.

In literature, overtaking decision problems have been addressed mainly from the perspective of lane-change decision-making. A lane-change has been generally considered as a maneuver to overtake a slow-moving vehicle ahead in multi-lane environments [6] suggested explicit decision rules for lane-change by investigating appropriate thresholds regarding the relative distances and velocities. In utility-based approaches [3,7,8,9], maneuvers were decided by scoring and comparing the efficiency of the traffic flow of the surrounding lanes [3] evaluated the desirability of a lane change in terms of the traffic situation of each lane such as average velocities and traffic densities [7] introduced the tactical behavior planning framework that considered velocity gains as important factors in evaluating the profitability of a lane change [9] suggested a probabilistic model to generate lane change proposals in unsatisfactory traffic situations such as following a slow preceding vehicle. Furthermore, recent studies [10,11,12] have adopted learning-based methods to predict the lane-change decisions of human drivers by learning from driving data. Overall, these decision-making approaches are established based on traffic-related factors such as traffic flow and spatial gaps, and seek an optimal maneuver to gain velocity [13,14,15]. In this aspect, stationary vehicles are commonly considered as obstacles to proceeding regardless of their potential movement. Unlike parked vehicles that are desired for drivers to overtake, there are many stationary vehicles on urban roads that should not be dismissed as obstacles. For example, a preceding vehicle temporarily stopped on urban streets is sometimes undesirable to overtake. Even though the conventional lane-change approaches contribute to generating overtaking decisions in terms of velocity gain based on the traffic situation, the approaches are not sufficient to handle stationary vehicles of which intentions should be considered in making overtaking decisions.

Recently, some previous studies have specialized in decision problems of overtaking stationary vehicles [16] suggested an obstacle detection algorithm using multiple sensors to discover roadside parked vehicles, followed by a decision-making model to overtake it with lateral movement. Similarly, in [17], an overtaking maneuver is made when the preceding vehicle is categorized as a parked vehicle through a Bayesian network-based classification algorithm. Furthermore, ref. [18] emphasized the importance of parking classification in maneuver decisions and contributed to analyzing important factors in inferring the parking intention of the preceding vehicle. Though these recent approaches focused on how to distinguish and avoid parked vehicles, overtaking maneuvers in autonomous driving are not necessarily limited to parking vehicles. In fact, human drivers often decide to overtake a preceding vehicle, which is stopped with an unaccountable reason, by considering the surrounding situation. Thus, the parking classification-dependent approaches have limitations to make human-like decisions especially in uncertain situations where the intention of the preceding vehicle is not sufficiently inferable.

To overcome the limitations of the previous studies, this paper proposes a learning-based decision-making system for overtaking stationary vehicles. This work aims to design and validate a decision-making system to generate human-like overtaking decisions in various urban traffic scenarios with stationary vehicles. The entire scheme of the proposed system is established based on Deep Neural Network (DNN) model in order to learn the decision strategy in complex situations from human demonstration data. In this scheme, the input of the DNN model needs to be carefully determined because it restricts how the model can interpret the perceived situation; otherwise, a large amount of data is required to automatically weigh the important factors from the raw input data. In the input selection process, we analyzed and selected significant factors which integrally involve the traffic-related and intention-related factors. This consequently enhanced the performance of the decision-making system to cope with various types of stationary vehicles on roads. To validate the performance, the proposed system was evaluated in various scenarios by comparing the results with the previous approaches.

This paper has been organized into the following contents. Section 2 describes the overall architecture of the proposed decision-making system. Section 3 explains the scene interpretation strategy by illustrating the procedure of the importance analysis on the decisions factors in overtaking maneuver decisions. Section 4 describes the proposed deep-learning-based decision-making model. The decision-making performance of the proposed system is evaluated in various traffic scenarios in Section 5. Finally, this paper is concluded in Section 6.

## 2. System Architecture

**Figure 2:**
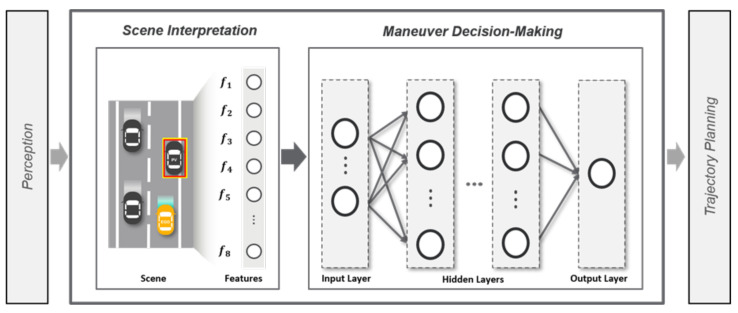
System architecture of the proposed decision-making system for overtaking maneuvers.

A decision-making system for overtaking maneuvers aims to generate behavioral decisions on whether to overtake the preceding vehicle in the driving situation. To fulfill this goal, the proposed decision-making system involves two sequential processes as shown in Figure 2. Scene interpretation process and maneuver decision-making process.

First, the driving scene needs to be interpreted in terms of the desirability of the overtaking maneuver. Decision factors that contain the information that should be utilized in making decisions are extracted from the entire perceived data in this process. Whereas some of the factors can be measured directly from in-vehicle sensors, there are factors that require additional calculation to estimate from the measurements. Thus, this scene interpretation process is responsible for post-processing the measured sensor data and estimating the values of the decision factors.

Second, a Deep Neural Network model is used to make a maneuver decision whether or not to overtake the preceding vehicle from the extracted factors. The DNN model is trained by demonstration data, which contains actual maneuver decision results of an expert driver in various situations. Since a DNN can model the nonlinear relationships between the input and the output, it is generally considered as a simple and effective method suitable for learning the complex policies of human drivers. The trained DNN model consequently predicts the maneuver decision of the driver in a certain situation, and the prediction result is used as an overtaking maneuver decision of the entire system.

## 3. Scene Interpretation for Overtaking Maneuver Decisions

Human drivers make maneuver decisions based on how they interpret the scene from what they perceive. To imitate the decision-making strategy of a driver by learning-based methods, it is important to figure out which factors in the perceived scene dominantly influence the driver’s decision. The importance of each factor can be estimated by analyzing the effect of the factor on the performance of the learning model. To analyze and select the optimal decision factors from all relevant factors, the series of processes is conducted as follows. First, the general characteristics of the human drivers’ overtaking maneuver decisions are examined with the domain knowledge in driving. Second, all relevant factors are listed up and categorized based on the general characteristics. Third, the dominant factors underlying a particular driver’s decisions are obtained by analyzing the importance of each factor in the decisions. Consequently, the obtained factors are considered as important factors in the scene interpretation, and thereby used as the input of the decision-making model.

### 3.1. General Characteristics

**Figure 3:**
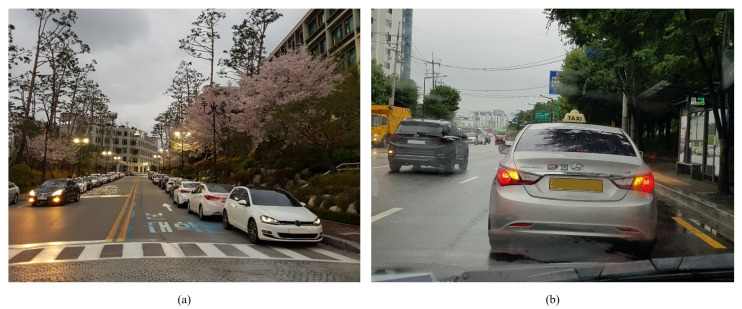
Stationary vehicles on road: (**a**) Illegally parked vehicles; (**b**) Stationary vehicle with an unpredictable reason.

There are three general characteristics of drivers’ decisions to overtake a stationary vehicle. First, when it is possible to clearly identify the reason why the vehicle is stopped on the road, the decision is made without much concern [18]. For example, since there are many parked vehicles along the curb, the drivers tend to decide to overtake the stationary vehicles which are off to the side of the road as shown in Figure 3a. Besides, the vehicle without any willingness to move forward, such as a vehicle with its door open, is easily considered as an obstacle to be overtaken. Thus, the intention of the stationary vehicle is one of the primary factors that influence the overtaking maneuver decisions.

Second, when it is difficult to correctly infer the intention of the preceding vehicle as shown in Figure 3b, drivers tend to make judgments based on the surrounding environments. For example, if there is no vehicle on the left lane, people do not hesitate to overtake the preceding vehicle regardless of its intention. In contrast, in situations where there are many vehicles nearby, drivers examine the surrounding situation to grasp the driving context and determine whether an overtaking maneuver is appropriate. Considering the fact that traffic engineering studies [19,20,21] insist drivers make decisions based on the spatial gaps and the traffic flow, this tendency is acceptable.

Third, the driving conditions of the ego vehicle may affect the decisions of the drivers. For example, if a driver discovers a stationary vehicle in the distance while driving at high speeds, it is sometimes preferred to steer slightly to the left to overtake the vehicle before getting closer to it. Additionally, the decision may change over time in the same situation. The increase in the time duration of waiting behind a stationary vehicle might induce a desire to escape from the situation, and the degree of tolerance depends on the aggressiveness of drivers [22]. These tendencies are meaningful factors in making decisions and vary from person to person even in the same situation.

To sum up, drivers commonly exhibit the characteristic of making situation-awareness decisions. This means that decisions depend not only on the intention of the preceding vehicle but also on traffic-related factors such as the states of the surrounding vehicles and the ego vehicle itself. The characteristics mentioned above need to be taken into account in selecting the decision factors to be used as the input of the decision-making model. Therefore, they are used as the basis for gathering relevant factors in the overtaking maneuver decisions.

### 3.2. Relevant Factors

#### 3.2.1. Categorization

**Figure 4:**
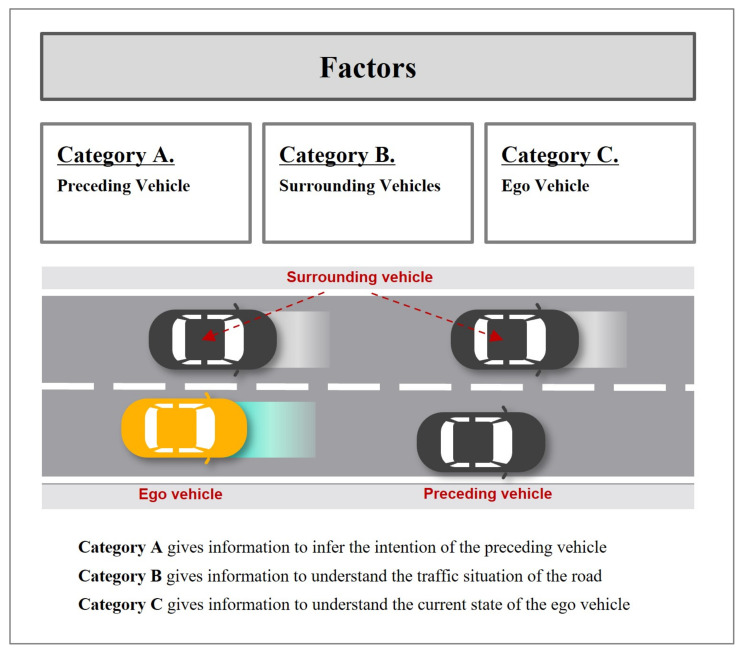
Categorization of the factors.

To collect relevant factors in a systematic manner, a categorization of factors is implemented in advance. The principle of categorization is inspired by the general characteristics of drivers mentioned previously. Thus, it aims to synthetically involve the intention-related factors and the traffic-related factors. Factors are finally divided into three groups as shown in Figure 4: factors regarding the preceding vehicle (Category A), surrounding vehicles (Category B), and the ego vehicle (Category C). Category A mainly includes factors to predict the future motion of the preceding vehicle whereas the rest categories have much to do with describing the entire traffic scene. All the relevant factors in each category are gathered and explained in the following section.

#### 3.2.2. List of Factors

- Category A. Preceding vehicle
  1 lateral distance to the right boundary of the road (’closeness to the curb’)
  2 lateral distance to the left boundary of the road (’room for overtaking’)
  3 duration of time it has been detected as stationary (’moving confidence’)
  4 velocity
  5 acceleration
  6 yaw angle
  7 yaw rate
  8 lane occupancy rate
  9 object width/length
- Category B. Surrounding vehicles
  - Nearest vehicle
    1 position
    2 velocity
    3 acceleration
  - Vehicles on surrounding lanes (Traffic flow)
    1 number of vehicles
    2 free space rate
    3 spatial gaps
    4 time/space mean speed
- Category C. Ego vehicle
  1 relative speed
  2 relative distance
  3 time-to-collision/time-headway (’collision risk’)
  4 waiting time duration

### 3.3. Factor Importance Analysis

All relevant factors are listed based on the general characteristics of human drivers. Even though these factors are expected to be relevant in general decisions, there might be less relevant or less influential factors in a certain driver’s decisions. It is necessary to select influential factors for the decision-making model to learn the driver’s policy efficiently. Therefore, each factor should be analyzed to confirm that it is beneficial to be used in the decision-making model.

In this work, we adopt a sequential backward feature selection (SBS) method [23] to analyze the importance of each factor in the model. This method estimates the importance of each factor according to the following sequence. At first, it starts evaluating the learning performance of a model that uses all the candidate factors as input. Then, by eliminating one factor out of the candidates, it evaluates the decrease in the learning performance. The degree of the decrease delineates the importance of the eliminated factor in the model. By iteratively estimating the importance of all the candidates, it is able to sort the candidates out according to importance. After removing the least important factor from the candidate list, the process is repeated until we get the rank of all the factors. The result of this method contains not only the rank of each factor but also the extent of the decrease in the learning performance when a factor is eliminated. If the decrease is almost zero or negligible, the eliminated factor would be considered as an irrelevant or redundant factor. In this way, the SBS method is utilized to analyze the importance of the factors and find the set of dominant factors to be used as model input by filtering out the insignificant factors.

## 4. Decision Strategy Based on DNN

### 4.1. Dataset

**Figure 5:**
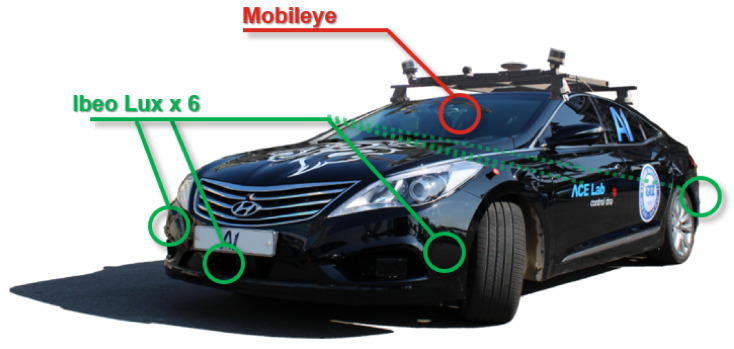
Sensor configuration of the test vehicle A1.

To train and evaluate the proposed system, we acquired the demonstration data by using the autonomous vehicle A1 of ACE Lab, Hanyang University. The sensor configuration of A1 is as shown in Figure 5. A1 is equipped with six LIDARs and a fusion box system (produced by IBEO), which are used to detect the surrounding vehicles. It also contains a Mobileye camera, which was mainly used to detect the lanes in this work.

**Figure 6:**
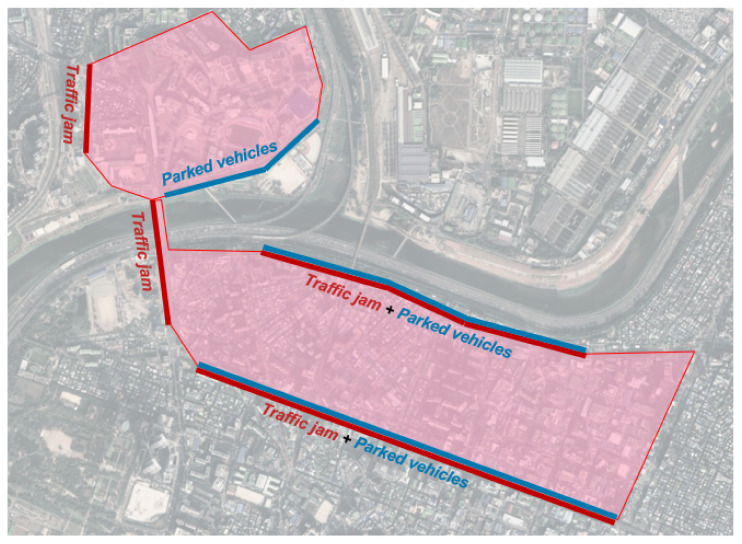
Target route for data acquisition.

To acquire the demonstration data, the test vehicle was driven by a certain driver, who is assumed to be an expert driver. The target route for the data acquisition is selected as shown in Figure 6, where various types of stationary vehicles exist. For example, there exist some vehicles stuck in traffic, along with some illegally parked vehicles. When the driver wanted to drive around a stationary vehicle while repeatedly operating the route, the driver transmitted a signal to indicate the decision by turning on the turn signal of the vehicle.

The collected driving data contains recorded scenes that are snapshots taken every 50 ms, which is the fixed sampling period. Each scene includes the perceived data and the decision signal of the driver. A scenario consists of a series of scenes, and the scenario can be classified into two categories: overtaking scenario and non-overtaking scenario. Although every scenario begins by detecting a preceding vehicle that is stationary regardless of the scenario category, how a scenario ends differs depending on the category. The not-overtaking scenarios usually end when the preceding vehicle starts moving and keep accelerating. On the other hand, the end of the overtaking scenarios is the moment when the driver begins executing the overtaking action; the execution procedure of the overtaking maneuver is not considered in this work.

**Figure 7:**
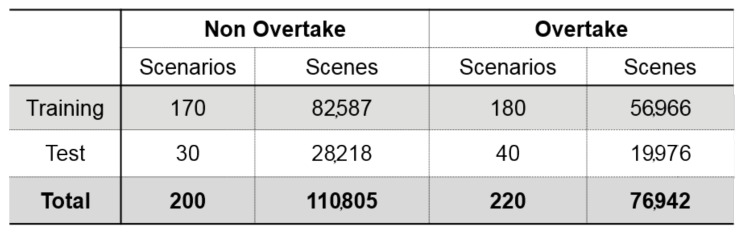
Demonstration dataset.

The summary of the acquired dataset is shown in Figure 7. It includes 200 non-overtaking scenarios and 220 overtaking scenarios. Each scenario varies in length, but they mostly range from 10 s to three minutes. Since the sampling period of the scene is determined as 50 ms, the number of scenes that each scenario contains ranges from 200 to 3600. Around 80% of the entire dataset is used as the training dataset, and the rest is used as the test dataset.

### 4.2. Input of the Model

**Figure 8:**
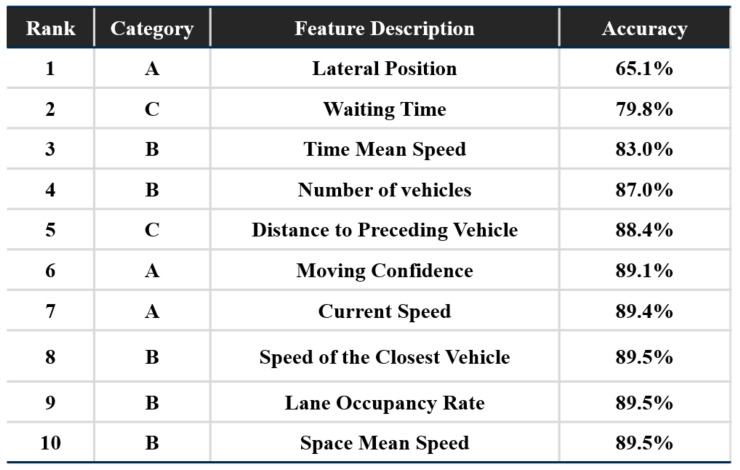
Results of the importance analysis.

The SBS method-based factor importance analysis method, illustrated in the previous section, is applied to find the dominant factors in learning a driver’s overtaking maneuver decisions. The results are shown in Figure 8, and there are some notable points on the results.

First, the most important factor in the decision-making model is the lateral position of the preceding vehicle which generally represents the closeness to the curb of the road. As closeness is a powerful feature mainly used in parking classification in literature, it gives substantial information about the intention of the preceding vehicle. Second, the waiting time of the ego vehicle in a given situation is selected as the second most important factor. This means that even if the situation is exactly the same, the decision of the driver may vary over time. If this factor is applied in the decision-making model, it is expected to be able to represent the driver’s individual characteristics such as aggressiveness or patience when driving. Third, the time-mean speed and the number of vehicles is selected as primary factors beyond many other factors in Category B. Since other additional factors such as space mean speed does not visibly improve the performance in the results, these two factors have been proven to be sufficient to interpret the surrounding traffic situation.

**Figure 9:**
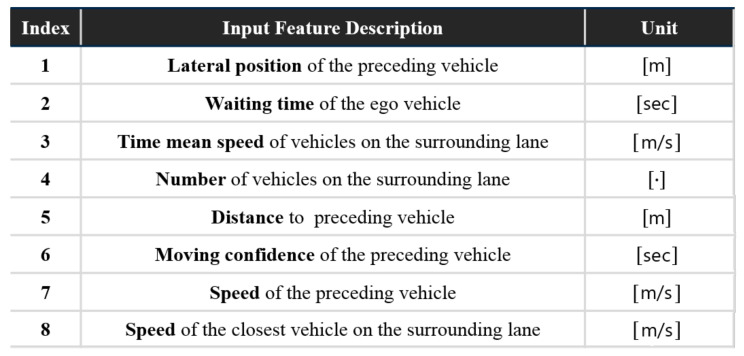
Input factors of the model.

Overall, the analysis results show that the intention-related factors in Category A and traffic-related factors in B or C are both influential in decision-making; thereby, they should collectively be extracted in the scene interpretation. According to the results, the eight most important factors are determined as the optimal input set of the proposed model in learning the driver’s decisions as shown in Figure 9.

The process of abstracting the factors from the perceived sensor data of the autonomous vehicle is carried out in the scene interpretation module. Details on how to calculate and extract factors in this module are given below; some of the factors that can be measured directly from in-vehicle sensors are excluded in the explanation.

**Figure 10:**
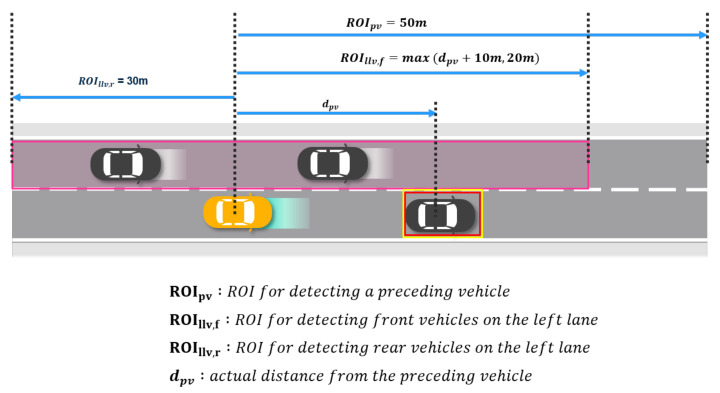
Region of interest in multi-lane environments. While ROIs for detecting a preceding vehicle and rear vehicles on the left lane are determined by the constant values, ROI for detecting front vehicles on the left lane depends on the distance to the preceding vehicle.

The time-mean speed and the number of vehicles in the neighboring lane are simply obtained by averaging the speed and counting the vehicles in the region of interest (ROI) on the left lane as shown in Figure 10. As the time-mean speed is the simple average of the spot speed, it is estimated by the number of vehicles in the ROI and the summation of the velocity of each vehicle. If there is no vehicle in the ROI, both factors are set as zero. The speed of the closest vehicle is obtained by finding a vehicle on the left lane that is of the shortest distance from the preceding vehicle. The velocity of the vehicle is used directly as the factor. If there is no vehicle in the ROI, the factor is set to zero as well.

There are two factors that contain temporal information: the waiting time of the ego vehicle and the moving confidence of the preceding vehicle. The former simply calculates the duration since the preceding vehicle in the ROI is detected as a stationary vehicle. This term only increases over time and does not stop or decrease in a scenario. On the other hand, the latter calculates the duration of which the preceding vehicle has been stationary. This term increases if the vehicle remains stationary, but may also drop to zero in the middle of the scenario if the vehicle begins moving. The difference between the two factors becomes obvious in the stop-and-go situations; the latter term would fluctuate whereas the former would keep increasing.

### 4.3. Training

The proposed model is trained by the acquired dataset. The input contains the data that is extracted from the recorded dataset by the scene interpretation module, and the output labels for training are the recorded binary decision signals of the driver. A cross-validation method has been used to prevent the overfitting problem of the trained model. For cross-validation, the training dataset is split into four parts so that three of them are used as the actual training set and the remaining one is used as the validation set. Then by repeatedly rotating the validation part four times, we are able to validate the model efficiently with a small amount of the dataset.

**Figure 11:**
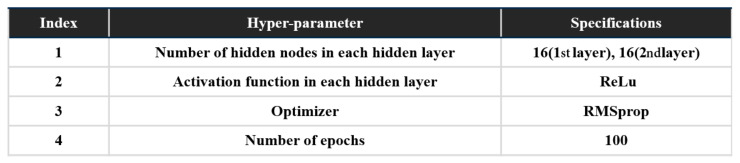
Optimized hyper-parameter set.

The performance when learning the complex relationship between the input and the output in the DNN model depends on the design of the hidden layers of the model. It is important to optimize the parameters of the hidden layers to fit into the particular problem to be solved, and this is conducted with various hyper-parameter optimization methods. In this research, hyper-parameters are optimized in the validation through the grid search algorithm. The results of the hyper-parameter optimization are shown in Figure 11; the number of hidden layers is pre-determined as two, having considered the size of the dataset and the dimension of the input data. With the optimized hyper-parameters, the DNN-based decision-making model is finally trained with the training dataset. The whole process of the training and validation utilized the open-source library Keras [24], which is widely used to develop a variety of deep learning models.

## 5. Evaluation

**Figure 12:**
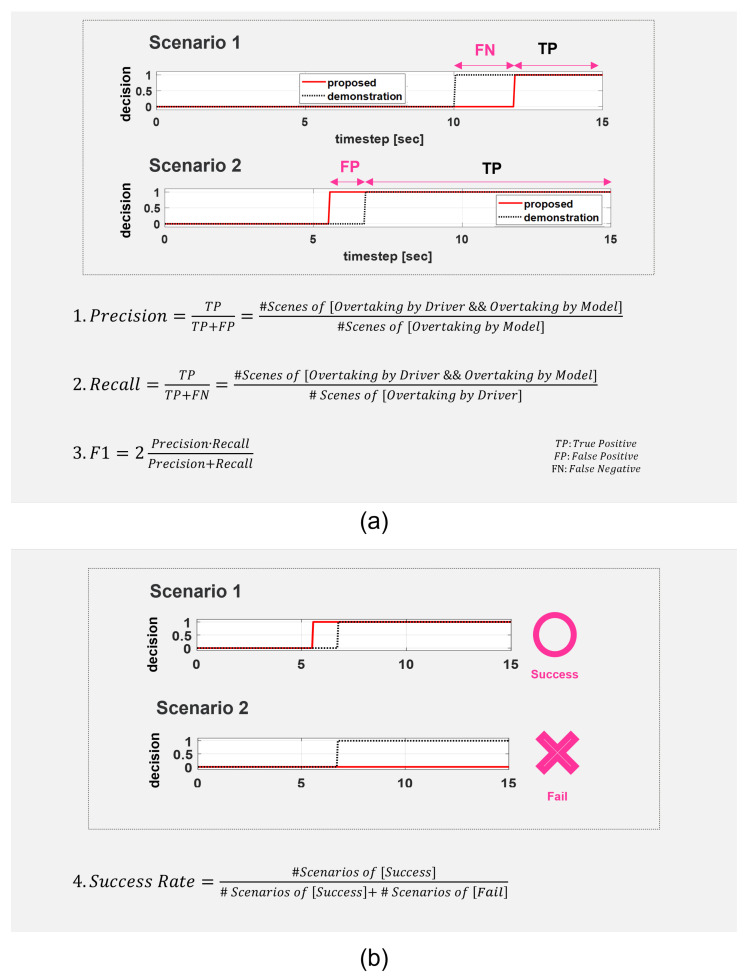
Evaluation metrics and their practical meaning: (**a**) Precision is the fraction of overtaking decisions executed by both the driver and the model among the overtaking decisions by the model. Recall is the fraction of overtaking decisions executed by both the driver and the model among the overtaking decisions by the driver. F1 is the harmonic mean of the precision and the recall; (**b**) Success rate is the fraction of success scenarios among the entire scenarios. A scenario is counted as a success scenario if the final decision of the driver and that of the model are identical.

We adopted several evaluation metrics which are the representative metrics for evaluating the prediction accuracy of the trained model: Precision, Recall, and F1 Score. The practical meanings of the metrics in this maneuver decision-making problem are explained in Figure 12a. The model performance was compared with those of other DNN models based on the previous approaches for validation. The DNN models are trained by using either the intention-related decision factors or the traffic-related factors respectively. Additionally, the performance of the proposed DNN model was compared with other models adopting other representative machine-learning techniques: Random Forest and KNN. Thus, the comparative evaluation aimed not only to verify the validity of the proposed decision factors in the learning performance, but also to demonstrate the superiority of the proposed DNN model as a decision-making system for overtaking maneuvers.

**Figure 13:**
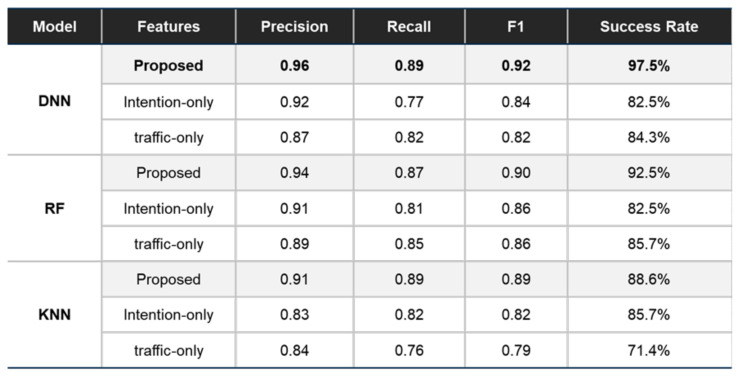
Quantitative evaluation results.

The evaluation of the proposed model and the other models was executed on the entire test dataset, of which acquisition environment was introduced in the previous section. The evaluation results are shown in Figure 13. The trained DNN, RF, and KNN models using the proposed decision factors showed improved learning performances in terms of Precision, Recall, and F1 scores compared to the models trained with either intention-only or traffic-only factors. Particularly in the results of the DNN models, the relatively low value of the Recall score of the intention-only or the traffic-only model represents the higher likelihood not to make overtaking maneuvers in the cases the human driver makes. On the other hand, since the proposed DNN model holds almost 90% of the overall performance, it verifies the point that the model properly reflects the characteristics of the driver in its overtaking maneuvers. Moreover, in the perspective of the learning methodology, the proposed DNN model had equal or higher scores on every metric than other learning models.

Since the three metrics evaluated the prediction accuracy at every scene, of which period is 50 ms, they showed the scene-wise performance of the learning model. To additively evaluate the scenario-wise performance, we defined a new metric which is Success Rate. As shown in Figure 12b, this compares the final decision of the model and that of the driver in a scenario. Regardless of the scene in which the overtaking maneuver of the model is made, the only matter of concern is whether or not the model makes an overtaking maneuver in the scenario. Though the metric lacks the ability to evaluate how similar the model is to the driver, it focuses on its ability to make proper decisions in a variety of scenarios.

The success rate of the proposed DNN model was 13–15% higher than the other DNN models, and 5–9% higher than other training models with the proposed factors. The higher success rate in the test datasets proved that the proposed model as a decision-making system was able to reflect the driver’s maneuver decisions. In fact, the test dataset includes various situations where the preceding vehicle is stationary for an uncertain reason. Thus, the proposed model for overtaking maneuvers was proven to be capable of handling uncertain situations in a similar way to the driver.

We further illustrate the decision-making results of the proposed system in some specific scenarios. The scenarios include various traffic situations. Through the presented scenarios, we aimed to analyze the influence of the selected decision factors and the ability of the proposed model.

### 5.1. Traffic Jam Scenario

**Figure 14:**
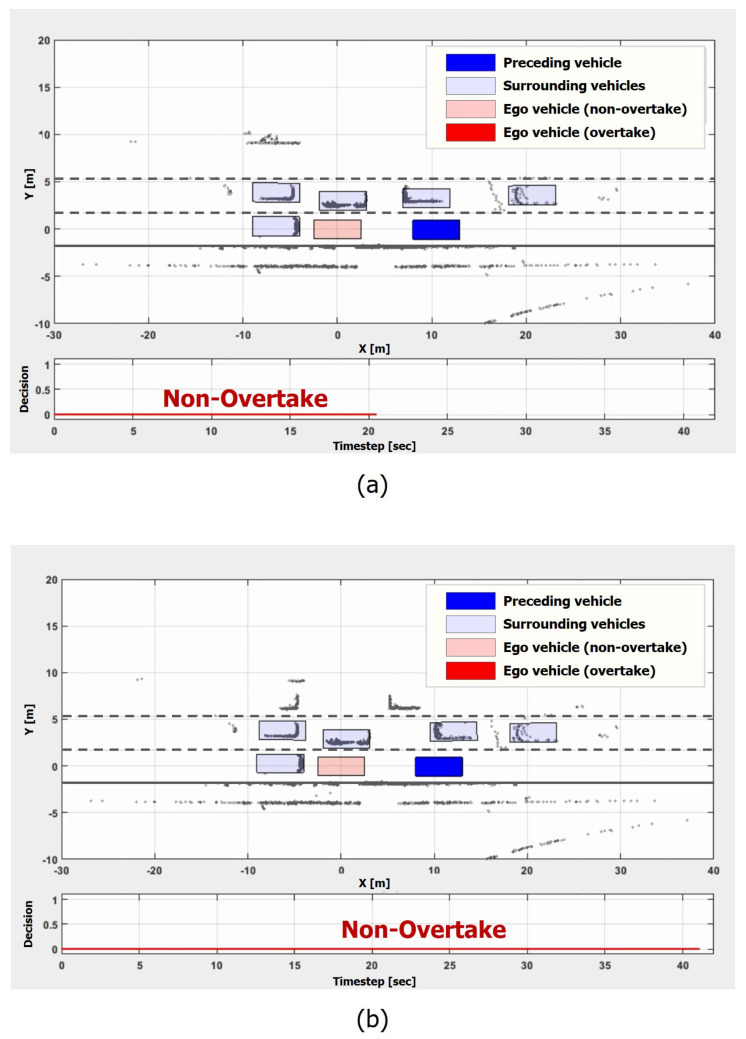
Traffic jam scenario. In the scenario, the ego vehicle is stuck in the middle of traffic where many vehicles are either stationary or moving extremely slowly: (**a**) t = 21 s; (**b**) t = 41 s.

We evaluated the proposed model in a non-overtaking scenario at first. Figure 14 depicts the scenes and the decision results of the proposed model at t = 21 s, and t = 41 s, respectively. In this scenario, the ego vehicle is stuck in the middle of traffic where many vehicles are either stationary or moving extremely slowly. This is one of the traffic jam situations where an overtaking maneuver is undesirable.

The proposed model did not make an overtaking decision within the whole scenario, as shown in Figure 14a,b. Though the ego vehicle had spent more than 40 s in the situation, the model kept its non-overtaking maneuver. This result shows that the model can recognize traffic jam situations and keep waiting behind stationary vehicles.

### 5.2. Light-Traffic-on-the-Left-Lane Scenario

**Figure 15:**
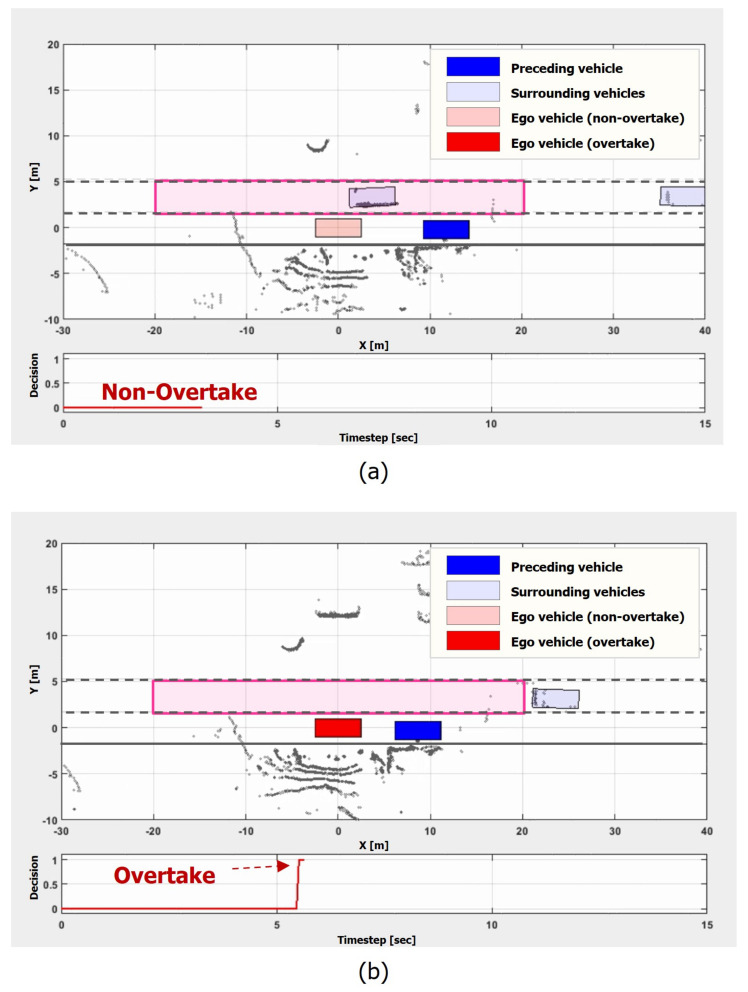
Light traffic scenario. In the scenario, the ego vehicle is behind a stationary preceding vehicle while the vehicles on the left lane are driving at around 30–40 km/h: (**a**) t = 3.5 s; (**b**) t = 5.5 s.

We evaluated the model in an overtaking scenario. The light traffic scenario in Figure 15 involves a preceding vehicle that is stationary and light traffic in the left lane. Since the preceding vehicle is stopped in the middle of the lane, the intention of the vehicle is hardly inferable in the scenario. As a result, the proposed model firstly kept the non-overtaking maneuver as the vehicle exists on the left lane in Figure 15a. However, at around five seconds as shown in Figure 15b, it decided to overtake when the number of vehicles on the left lane dropped to zero. This result shows that the proposed model properly makes an overtaking maneuver decision by considering the surrounding traffic situation.

### 5.3. Heavy-Traffic-on-the-Left-Lane Scenario

**Figure 16:**
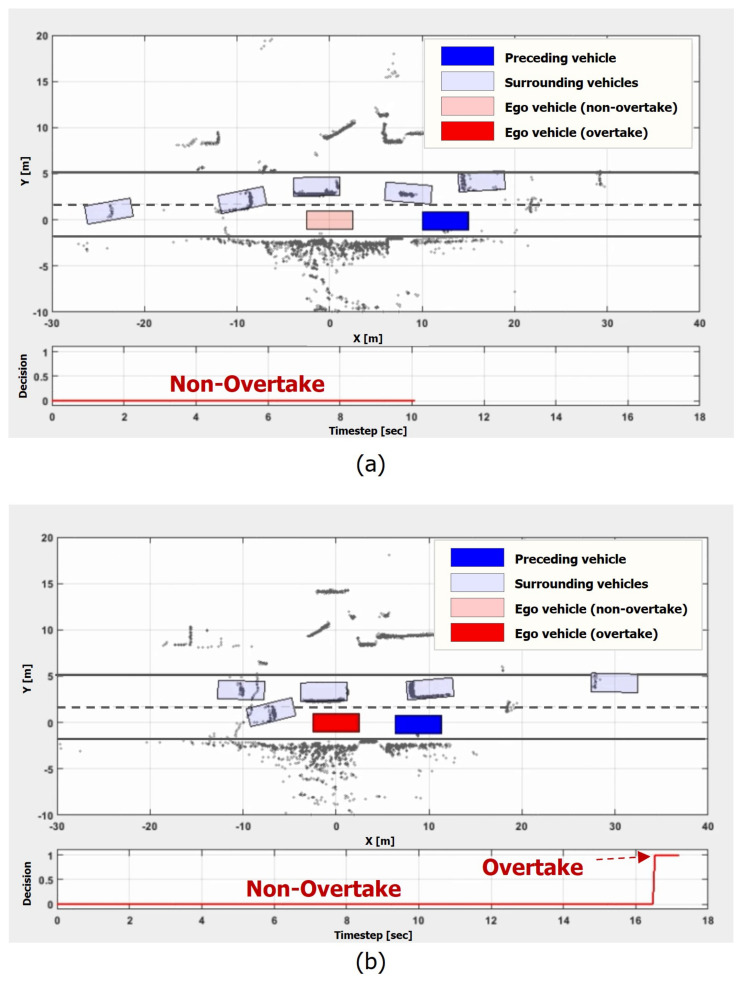
Heavy traffic scenario. In the scenario, the ego vehicle is behind a stationary preceding vehicle while the vehicles on the left lane are driving at around 10–20 km/h: (**a**) t = 10 s; (**b**) t = 17 s.

The third scenario also involves a preceding vehicle stopped in the middle of the lane. The difference of this scenario from the previous one lies in the traffic flow of the left lane. As in Figure 16, there are many slow-moving vehicles on the left lane.

Figure 16a,b shows that the proposed model did not make an overtaking maneuver at t = 10 s, but it decided to overtake the preceding vehicle at t = 17 s. The results demonstrate two important points. First, the proposed model was able to distinguish the heavy traffic scenario from the previous traffic jam scenario. The consideration of the traffic flow of the surrounding lanes facilitated appropriate overtaking decisions. Second, the time duration was considered in the decision. After waiting about 17 s in almost the same situation, the model changed its maneuver. Therefore, the results of the scenario verified the ability of the proposed model to integrate various factors from the scene and make proper maneuver decisions in uncertain situations.

## 6. Conclusions

This paper addressed the limitations of the previous approaches for overtaking maneuver decision-making systems, which have highly depended either on spatial gap-oriented lane-change decisions in multi-lane environments or parking classification of stationary vehicles. The paper presented a DNN-based decision-making system that can be utilized in generalized situations by learning the strategy of a human driver to navigate complex driving situations. The entire system consists of two modules: Scene interpretation and maneuver decision-making module. In the scene interpretation module, the dominant decision factors for the driver’s decisions, which integrally contain intention-related and traffic-related information, are extracted. The selected factors are used as the input of the DNN-model, and the model is trained by the driver’s demonstration data. The trained model is expected to make human-like overtaking maneuver decisions in generalized situations, including the situation where the preceding vehicle is stationary with an uncertain intention.

The training results were analyzed and compared with the trained models representing previous approaches. The learning performance of the proposed DNN model was higher in all metrics than not only the DNN models trained by either intention-related or traffic-related decision factors but also other learning models utilizing the proposed decision factors. It showed that the proposed DNN model is more suitable to predict the overtaking maneuver decisions of the driver. Furthermore, we analyzed the decision-making results of the system in various traffic scenarios for evaluation. The experimental results in the presented scenarios verified that the proposed model decides to overtake a stationary vehicle by considering not only the intention of the preceding vehicle but also the traffic situations from the perceived scene. Overall, the proposed system showed 97.5% of the successful decision-making results in the entire scenarios. The results demonstrate convincingly that the proposed decision factors and the trained model contributed to making more human-like overtaking maneuver decisions in various scenarios than the previous approaches.

Although the proposed model showed the improvement of the decision-making performance in uncertain situations compared to the previous approaches, there remains the necessity of further works. By acquiring more data, the designed model should be trained and validated for more reliability. Furthermore, by utilizing the demonstration data from multiple drivers, the research should involve evaluating the capability of the proposed model to reflect the characteristics of the individual drivers.

## Data Availability

Due to the nature of this research, participants of this study did not agree for their data to be shared publicly, so supporting data is not available.
